# The influence of thermal tempering on the fracture resistance, surface microstructure, elemental surface composition, and phase analysis of four heat-pressed lithia-based glass ceramic crowns

**DOI:** 10.1186/s12903-025-05509-1

**Published:** 2025-02-05

**Authors:** Khaled Nasser, Amr EL-Etreby, Soha Osama Nabih

**Affiliations:** 1 Department of Fixed Prosthodontics, Faculty of Dentistry, Delta University, Mansoura, Egypt; 2 Department of Fixed Prosthodontics, Faculty of Dentistry, Ain Shams University, Cairo, Egypt

**Keywords:** Pressable ceramics, Lithium disilicate, Lithium silicate, Fracture resistance, Thermal tempering

## Abstract

**Background:**

This in-vitro study aimed to evaluate the impact of thermal tempering and ceramic type on the fracture resistance, surface microstructure, elemental surface composition and phase analysis of four heat-pressed glass ceramics.

**Methods:**

A total of 84 glass-ceramic crowns were pressed and randomly allocated into four equal groups (*n* = 21) according to the ceramic type: Group (E): IPS e.max Press, Group (L): GC initial LiSi Press, Group (C): Celtra Press and Group (A): VITA Ambria. The crowns of each group were equally allocated into three subgroups (*n* = 7) regarding the subsequent thermal tempering temperature. Subgroup (T0): No tempering. Subgroup (T1): Tempering at 9% below pressing temperature. Subgroup (T2): Tempering at 5% below pressing temperature. Samples were tested for fracture resistance using a universal testing machine. A scanning electron microscope, X-ray diffraction, and Energy Dispersive x-ray analysis were utilized to disclose the microstructural features.

**Results:**

When there is no tempering, IPS e.max press showed a significant elevated fracture resistance (P-value = 0.002). There was an insignificant difference between other ceramics. While with tempering (T2) as well as (T1), Lisi press (L) showed a significant elevated fracture resistance. There was an insignificant difference between other ceramics (P-value = 0.004).

**Conclusions:**

Incorporation of zirconia oxide into the lithium disilicate glass matrix did not show improvement in the fracture resistance. Thermal tempering procedure had significant effect on fracture resistance. Thermal tempering technique had no influence the elemental surface composition and phase analysis yet T2 samples showed changes in crystal size and orientation.

## Background

All-ceramic restorations have been very popular in the last decades because of the applications of modern dental technologies and the improvement in the mechanical, aesthetic and biocompatible properties of these materials [1, 2]. The development of dental ceramics with high flexural strength, high fracture toughness, high aesthetic and high chemical resistance is a formidable challenge in dental industry [2–4]. Recently novel ceramic materials have been developed in an effort to combine polycrystalline ceramic mechanical qualities and glass matrix ceramics superior esthetics [5, 6].

Lithium disilicate glass–ceramic (IPS e.max, Ivoclar Vivadent, Schaan, Liecht-enstein) was introduced into the dental practice in 1998 [7–9]. It was produced with excellent physical properties and exceptional translucency by using different firing processes [10–12]. Lithium disilicate crowns can be pressed using IPS e.max Press ingots or milled using IPS e.max CAD blocks [13, 14]. Crystals of IPS e.max Press reaching 3 to 6 μm in length and comprising roughly 70% of its microstructure are embedded in a glassy matrix [15, 16]. It has a 400 Mpa flexural strength. However, this material’s strength is still insufficient for usage in high-stress bearing areas [17, 18].

GC LiSi press (GC Initial, Tokyo, Japan), the first lithium disilicate ceramic with High Density Micronization (HDM), was released to the market [19, 20]. According to the company, their specific technology offers remarkable physical qualities and the most natural and true aesthetic [21]. Its flexural strength is > 500 MPa, wear-resistant, and stable after multiple firings [21]. Zirconia-reinforced lithium silicate (ZLS) ceramic (Celtra press, Sirona Dentsply, Milford, DE, USA) is an alternate method to increase translucency and strength [22, 23]. It consists of a vitreous matrix, a crystal structure comprised of lithium silicate crystals with tetragonal zirconia reinforcements (about 10% by weight) [24]. ZLS ceramic is a promising restorative material that exhibits unique behavior upon aging. Their physical, chemical and mechanical qualities can be adjusted by varying the structure and heat treatment process [17]. According to the manufacturer, these materials offer flexural strength > 500 MPa after power glazing. Compared to lithium disilicate glass-ceramic, it exhibited a lower brittleness index, higher machinability, high edge strength, and better surface finish [25–27].

In a clinical context, the ability of dental crowns to withstand the stresses from mastication and other forces is crucial [28]. Unlike flexural strength, which measures material performance under bending loads, fracture resistance more directly reflects how a restoration will behave under real-world conditions, including uneven forces. Fracture resistance encompasses various modes of failure that a crown may encounter in the oral environment [29]. Several factors can influence the results of the fracture resistance test, including the material’s composition, mechanical characteristics, and the applied load on the restoration. The fracture resistance test can assist in identifying the force that could break the tooth‒restoration complex, thereby suggesting optimal preparation designs and restorative materials with the greatest resistance to fracture. Lithium disilicate crown’s fracture resistance is affected by a variety of parameters, including ceramic material composition, ceramic microstructure, crown thickness, and thermal tempering [8, 22, 30].

Sieper K et al. [31] evaluated the fracture strength of lithium disilicate crowns compared to zirconia reinforced lithium silicate crowns. They concluded that thin crowns of the tested materials showed fracture strengths of 2,000 N or even higher after cyclic loading. The mean fracture strength of thin lithium disilicate crowns showed a significantly higher fracture strength than zirconia-reinforced lithium silicate crowns.

Hot pressing has been a preferred method for the manufacturing of glass-ceramic restorations because hot-pressed glass-ceramic restorations offer superior fitting precision, edge quality, minimal porosity, and great mechanical strength in comparison to computer-aided design-computer-aided manufacturing (CAD/CAM) milled materials [32–34]. Because ingot shades are restricted, heat-pressed glass ceramic restorations may need extra firing cycles to get a more aesthetically pleasing outcome, whether for glazing, specific dying, or surface veneering [35, 36]. Changes in the mechanical and optical characteristics of glass ceramic restorations caused by heat treatments have an impact on the final clinical outcomes [37, 38].

Thermal tempering is a technique whereby a heat-pressed ceramic crown is heated to a temperature just above the glass transition region, yet below its softening point [39]. Abo-Elezz et al. [40], reported that heat tempering with a temperature 5% below pressing temperature increased the biaxial flexural strength of different lithia based glass ceramic discs and their phase analysis results showed no change in the main crystalline phase. Thermal tempering technique can influence the crystal size and its morphology [41]. The growth of the grain size indicates that the crystallization process continues during the tempering procedure and more lithium silicate crystals can be precipitated. This crystallization process deposited more crystals in the glassy matrix and increased the interlocking effect leading to an increase in the fracture resistance [42].

The manufacturer of a newly introduced zirconia-reinforced lithium disilicate glass-ceramic (VITA Ambria, VITA Zahnfabrik, Germany) claimed that the thermal tempering cycle at 9% below pressing temperature could raise the material’s flexural strength from 400 MPa after pressing to 550 MPa [8, 43–45]. The thermal tempering procedure has a considerable effect on the shape and size of the crystal. The increase in grain size indicates that the crystallization process continues during thermal tempering, causing the precipitation of more crystals and a rise in fracture resistance [46, 47].

Oh et al. [48], concluded that heat tempering of IPS Empress 2 had an increased flexural strength of 387 MPa and their Scanning Electron Microscope results (SEM) showed highly packed, interlocking microstructure of many lithium disilicate crystals in the glass matrix. Also, El-Etreby et al. [39], concluded that the biaxial flexural strength of heat pressed lithium disilicate glass ceramics had increased after heat tempering and this result was explained by the significant increase of crystal size after heat tempering. Hallmann et al. [21], found that Celtra press had the lowest biaxial flexural strength values after heat tempering at 860^ο^C when compared to IPS e.max press and Initial LiSi press. Sun et al. [49], found that the flexural strength was increased when Lithium disilicate (IPS e.max press) glass ceramics were subjected to heat tempering at 820^ο^C, this increase in flexural strength was attributed to the change in the crystals morphology from spherical to rod shaped.

Currently, there is no consensus in the literature on the influence of thermal tempering protocol on the fracture resistance of various lithia-based glass ceramic restorations. Therefore, this in-vitro study aimed to assess the effect of thermal tempering protocol and ceramic type on the fracture resistance of four heat-pressed glass ceramics. The null hypothesis of this study postulated that neither the type of heat-pressed ceramic nor the thermal tempering protocol would impact the fracture resistance of the ceramic material tested.

## Materials and methods

### Materials

Several types of lithium disilicate and zirconia-reinforced lithium silicate materials, were used in this study as shown in Table 1. IPS e.max press (Ivoclar vivadent, Schaan, Liechtenstein) consists of approximately 70% lithium disilicate crystals embedded in a glass matrix. GC Initial LiSi press (GC Initial, Tokyo, Japan) features 0.5 to 1.5 μm micro-crystals dispersed homogeneously in the glass matrix, also utilizing HDM to enhance material performance, while Celtra press (Sirona Dentsply, Milford, DE, USA) incorporates 10% zirconia with nano-scale lithium phosphate, providing improved mechanical properties, and Vita Ambria (VITA Zahnfabrik, Germany) combines 10% zirconia with lithium disilicate crystals of 2.5 to 3.5 μm, offering a strong, durable material with complete zirconia dissolution in the glass phase.

**Table 1:** Materials used in this study

Material	Trade name	Manufacturer	Description
Lithium disilicate	IPS e.max press	Ivoclar vivadent, Schaan, Liechtenstein	lithium disilicate crystals (approx. 70%), Li_2_Si_2_O_3_, embedded in a glassy matrix.
High Density Micronization (HDM) Lithium disilicate	GC initial LiSi press	GC Initial, Tokyo, Japan	0.5 to 1.5 μm micro-crystals are equally dispersed within the glass matrix for a homogeneous fill and a High Density Micronization (HDM)
Zirconia reinforced lithium silicate	Celtra press	Sirona Dentsply, Milford, DE, USA	1.5 μm plus nano-scale lithium phosphate, Li_2_O and SiO_3_ and 10% zirconia (ZrO_2_), which is completely dissolved in the glass phase
Zirconia reinforced lithium disilicate	Vita Ambria	VITA Zahnfabrik, Germany	2.5 to 3.5 μm Li_2_O and SiO_2_ and 10% zirconia (ZO_2_), which is dissolved completely in the glass phase

### Methods

**Fig. 1:**
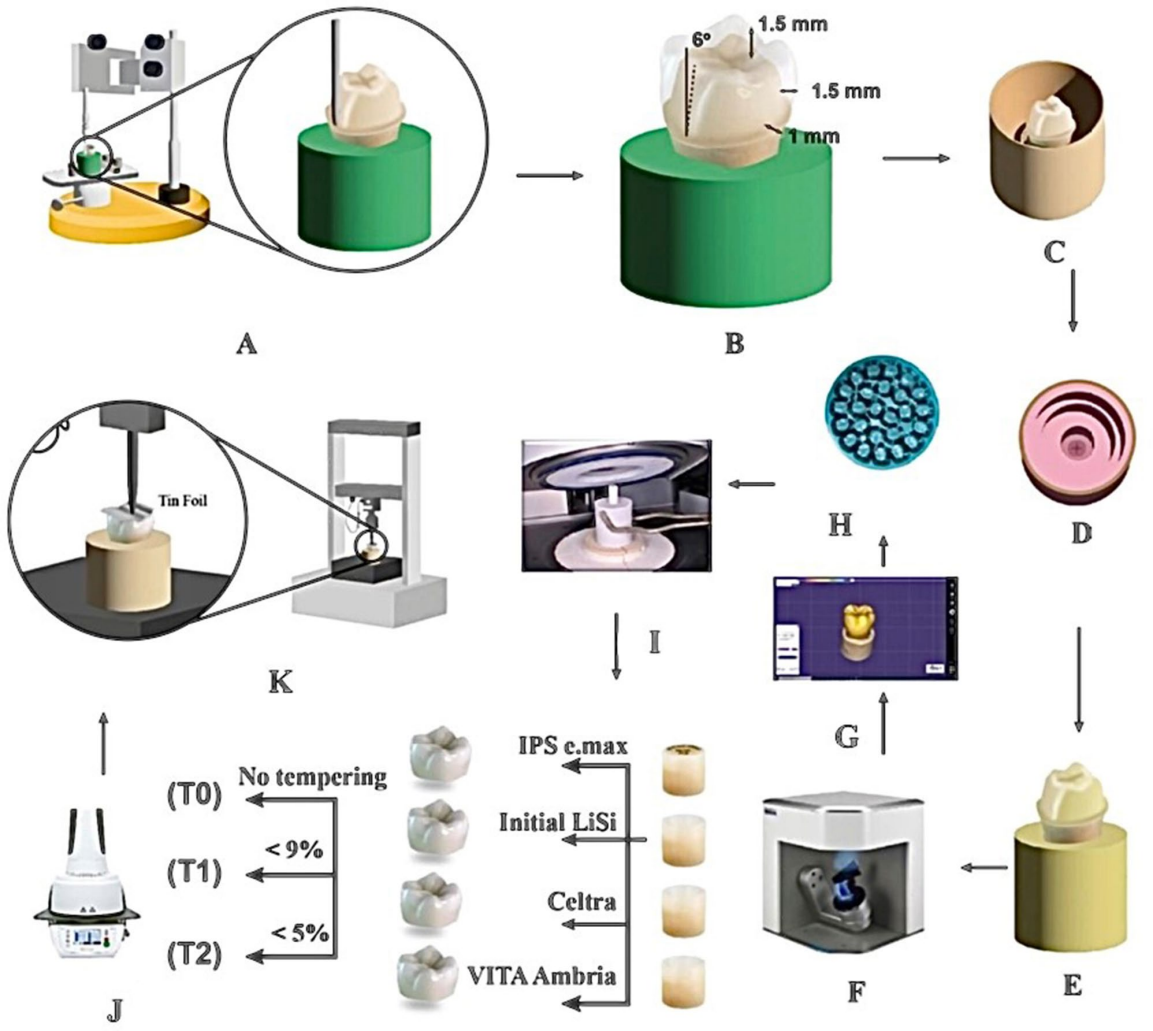
Schematic presentation of the specimens’ preparation and the fracture resistance test. **A** Surveyor guided preparation of mounted lower first molar acrylic prototype. **B** Preparation criteria for an all-ceramic posterior crown. **C** Preparation of the master model for duplication procedure. **D** Dulplicating mold. **E** Epoxy resin replica. **F** Prepared die scanning using Identica blue scanner. **G** Virtual model of restoration design. **H** Milled wax patterns. **I** Heat pressing using four different lithium disilicate ingots. **J** Different thermal tempering protocol applied for each material. **K** Fracture resistance testing using universal testing machine

A schematic presentation of the specimens’ preparation and the fracture resistance test is presented in Fig. 1.

### Sample size calculation

A power analysis was designed to have adequate power to apply a statistical test of the null hypothesis that there is no difference would be found regarding fracture resistance. By adopting alpha (α) and beta (β) levels (0.05) (i.e., power = 95%), and an effect size (f) of (1.02) calculated based on the results of a previous study [42]; the total minimum required sample size (n) was found to be (36) samples (i.e.,9 samples per group and 3 samples per subgroup). Sample size calculation was performed using R statistical analysis software version 4.3.2 for Windows^1^.

### Epoxy resin die production

A lower first molar acrylic prototype was mounted in acrylic resin base as a mold, Then the tooth was mounted with the aid of surveyor. Computer numerical control lathe-cut milling machine (C.N.C premium 4820, imes-icore Eiterfeld, Germany) was utilized to prepare an acrylic lower first molar for a posterior crown with 1.5 mm of occlusal reduction and 1 mm of axial reduction. A 1 mm circumferential supragingival heavy chamfer finish line was created. Impressions of the master model were made using silicon duplication material (Replisil 22 N, dentecon, Germany). The molds were then poured with chemically cured epoxy resin (Chemapoxy 150, MBC, Egypt) to produce 84 epoxy resin dies.

### Fabrication of CAD wax patterns

To standardize the crown’s anatomy, shape, and thickness, the preparation was scanned by Identica blue scanner (MEDIT corp., Seoul, Korea), and then the data was transferred to an Exocad computer software version 2017 (Exocad GmbH, Darmstadt, Germany) to design the crowns. The crown design was a duplicate of pre-preparation scan. A 5-axis milling machine (VHF, CAM 5-S1, Germany) was used for milling of wax patterns from wax blocks (YAMAHACHI, Japan). A total of 84 wax patterns were milled and checked for adaptation over their corresponding dies.

### Samples grouping

The wax patterns were then allocated into four groups (*n* = 21) according to the type of heat pressed glass ceramic material and each group was further subdivided into three subgroups (*n* = 7) according to the subsequent thermal tempering temperature as shown in (Table 2).

**Table 2:** Sample grouping

Material	Group (E) (*n* = 21) IPS e.max press	Group (L) (*n* = 21) GC initial LiSi press	Group (C) (*n* = 21) Celtra press	Group (A) (*n* = 21) VITA Ambria	Total
Thermal tempering					
Without thermal tempering (T0)	ET0 (*n* = 7)	LT0 (*n* = 7)	CT0 (*n* = 7)	AT0 (*n* = 7)	28
Thermal tempering at 9% below pressing temperature (T1)	ET1 (*n* = 7)	LT1 (*n* = 7)	CT1 (*n* = 7)	AT1 (*n* = 7)	28
Thermal tempering at 5% below pressing temperature (T2)	ET2 (*n* = 7)	LT2 (*n* = 7)	CT2 (*n* = 7)	AT2 (*n* = 7)	28
Total	21	21	21	21	84

### Fabrication of heat-pressed ceramic crowns

All wax patterns were sprued and invested following the manufacturer’s guidelines. Phosphate bonded investment material (Ivoclar Vivadent, Zurich, Switzerland) was mixed in a vacuum mixer for 40 s (Whip Mix Corp.,Kentucky, USA). Wax removal was conducted using a wax burn-out furnace (Ney, US Dental Depost, USA) following the manufacturer’s instructions. The ring was inserted and left for 60 min at 850 °C. After removing the investment ring from the burnout furnace, each ceramic ingot was inserted into the investment ring, and then the alox plunger was positioned (Ivoclar vivadent, Schaan, Liechtenstein). Each ceramic material firing program was selected according to the manufacturer’s guidelines and activated. After the program was completed, the investment ring was removed from the heat press furnace and left to be cooled to room temperature on a wide-meshed grid. The pressed crowns were unsprued, finished, and polished with low-speed fine finishing stones (Polishing kit, Dentsply, Germany) after being divested.

### Thermal tempering protocol

For subgroups T1 and T2, thermal tempering protocols were applied. The thermal tempering involved applying heat treatments to the selected ceramic materials at temperatures below their softening point. Ceramic crowns were first pressed at their maximum pressing temperature, and then subjected to thermal tempering at two different temperature conditions: 9% below the maximum pressing temperature (T1) and 5% below the maximum pressing temperature (T2). The tempering temperature was calculated for each group based on its maximum pressing temperature. The calculated thermal tempering temperature for each ceramic is presented in (Table 3).

**Table 3:** Comparison between pressing temperature and tempering temperature of each lithia-based glass ceramic material

Material	Maximum pressing temperature without tempering (°C) (T0)	Calculated tempering temperature at 9% below maximum pressing temperature (°C) (T1)	Calculated tempering temperature at 5% below maximum pressing temperature (°C) (T2)
Group (E)	917	834	871
Group (L)	910	828	864
Group (C)	865	787	822
Group (A)	890	800	846

### Restoration cementation

All crowns were cemented using dual cure resin cement (Duo-Link, BISCO, USA). Using light finger pressure, each crown was seated on its corresponding die with light finger pressure. Using loading equipment, a 5 kg axial force was applied for 10 min. The margins were light-cured for three seconds using light-curing device (SDI, Australia). The excess luting material was then eliminated. Then light curing for the luting agent was carried out for 20 s per surface.

### Fracture resistance testing

Using a universal testing equipment with a 5 kg load cell and a computer software, data were collected (Instron 3345, Instron, USA). Compressive mode of load was introduced to the center of the occlusal surface, with the load applicator tip only touching the buccal incline of the lingual cusp and lingual incline of the buccal cusp, using a metallic rod with a spherical tip (5.6 mm in diameter) and a tin foil sheet to achieve homogenous stress distribution and minimize the transmission of localized force peaks. The failure load was audibly signaled by a crack and confirmed by a dramatic decrease in the load-deflection curve captured using computer software. Finally, the fracture load was measured in Newtons, and the mechanism of failure for each restoration was determined.

**Failure modes** were observed by the same operator, classified and tabulated into 3 groups: Type I: Cracking, Type II: Chipping or partial fracture, and Type III: Catastrophic or fragmented fracture.

### Scanning electron microscope (SEM)

The microstructure of a broken fragment of each glass-ceramic was determined by scanning electron microscopy (SEM) (DSM 962 device, Zeiss, Germany) at magnification of 15,000X. For the SEM images, the specimens were examined three times: after pressing (T0), (T1) and (T2) thermal tempering. The specimens were washed using acetone and distilled water. Next, they were placed in an ultrasonic bath at room temperature for 10 min. Then, they were imaged under SEM after being sputter-coated with gold.

### Xray diffraction test (XRD)

Fragments of one sample from each subgroup were exposed to XRD (Xpert pro, USA) to determine the crystalline phases. Samples were placed on the holder of the diffractometer and scanned using Cu K α x-ray angle from 4 to 80 degrees, 2θ with a step size of 0.04 degrees and 5 s-step intervals.

### Energy dispersive x-ray analysis (EDAX)

One sample from each subgroup was examined for elemental surface micro analysis using Energy Dispersive X-ray Analysis (FEI Czech SEM USA). The elemental composition of each ceramic subgroup was calculated and tabulated.

### Statistical analysis

Statistical analysis was performed with R statistical analysis software version 4.1.3 for Windows (R Core Team, Vienna, Austria). Shapiro-Wilk’s test was used to test fracture resistance data for normality. Homogeneity of variances was tested using Levene’s test. Data showed parametric distribution and variance homogeneity and were analyzed using two-way ANOVA. Comparison of simple main effects was done utilizing the error term of the two-way model with p-values adjustment using Bonferroni correction. The significance level was set at *p* < 0.05 within all tests. Categorical data was presented as frequency and percentage value and was analyzed using Fisher’s exact test.

## Results

### Fracture resistance test

#### Comparison between ceramic types

##### When no thermal tempering was applied (T0)

There was a statistically significant difference between ceramic types (*P*-value = 0.002, Effect size = 0.181). Pair-wise comparisons between ceramic types revealed that IPS e.max (E) showed the statistically significantly highest mean fracture resistance. There was no statistically significant difference between LiSi press (L), Celtra press (C) and Vita Ambria (A); all showed statistically significantly lower mean values.

##### While with thermal tempering (T2) as well as (T1)

There was a statistically significant difference between ceramic types (*P*-value = 0.004, Effect size = 0.166) and (*P*-value < 0.001, Effect size = 0.23), respectively. Pair-wise comparisons between ceramic types revealed that LiSi press (L) showed the statistically significantly highest mean fracture resistance. There was no statistically significant difference between IPS e.max press (E), Celtra press (C) and Vita Ambria (A); all showed statistically significantly lower mean values as shown in Table 4.

**Table 4:** Comparison between fracture resistance (N) values of ceramic types with each tempering temperature

Thermal tempering	IPS e.max (E) (*n* = 7)		LiSi press (L) (*n* = 7)		Celtra press (C) (*n* = 7)		Vita Ambria (A) (*n* = 7)		*P*-value	Effect size (Partial eta squared)
	Mean	SD	Mean	SD	Mean	SD	Mean	SD		
T0	1611.1 ^A^	98.1	1367.5 ^B^	106.9	1312.1 ^B^	141.3	1203.4 ^B^	133	0.002*	0.181
T1	1754.9 ^B^	210.8	2200 ^A^	232.3	1951.7 ^B^	151.5	1802.1 ^B^	163.4	< 0.001*	0.23
T2	2245.8 ^A^	156	1951.2 ^B^	156.6	2022.7 ^B^	365.5	1862.3 ^B^	285.5	0.004*	0.166

*: Significant at *P* ≤ 0.05, Different superscripts in the same row indicate statistically significant difference between ceramic types

#### Comparison between thermal tempering temperatures

##### Whether with LiSi press (L), Celtra press (C) as well as Vita ambria (A)

There was a statistically significant difference between thermal tempering temperatures (*P*-value < 0.001, Effect size = 0.548), (*P*-value < 0.001, Effect size = 0.432) and (*P*-value < 0.001, Effect size = 0.397), respectively. Pair-wise comparisons revealed that there was no statistically significant difference between (T2) and (T1) thermal tempering; both showed statistically significantly higher mean fracture resistance than (T0).

##### While with IPS e.max (E)

There was a statistically significant difference between thermal tempering temperatures (*P*-value = 0.008, Effect size = 0.126). Pair-wise comparisons revealed that there was no statistically significant difference between (T0) and (T1) tempering; both showed statistically significantly lower mean fracture resistance than thermal tempering (T2) as shown in Table 5.

**Table 5:** Comparison between fracture resistance (N) values of tempering temperatures with each ceramic type

Ceramic	(T0) (*n* = 7)		(T1) (*n* = 7)		(T2) (*n* = 7)		*P*-value	Effect size (Partial eta squared)
	Mean	SD	Mean	SD	Mean	SD		
IPS e.max (E)	1611.1 ^B^	98.1	2200 ^A^	232.3	2245.8 ^A^	156	0.008*	0.126
LiSi press (L)	1367.5 ^B^	106.9	1754.9 ^B^	210.8	1951.2 ^A^	156.6	< 0.001*	0.548
Celtra press (C)	1312.1 ^B^	141.3	1951.7 ^A^	151.5	2022.7 ^A^	365.5	< 0.001*	0.432
Vita Ambria (A)	1203.4 ^B^	133	1802.1 ^A^	163.4	1862.3 ^A^	285.5	< 0.001*	0.397

*: Significant at *P* ≤ 0.05, Different superscripts in the same row indicate statistically significant difference between tempering temperatures

### Mode of failure of fractured samples

**Fig. 2:**
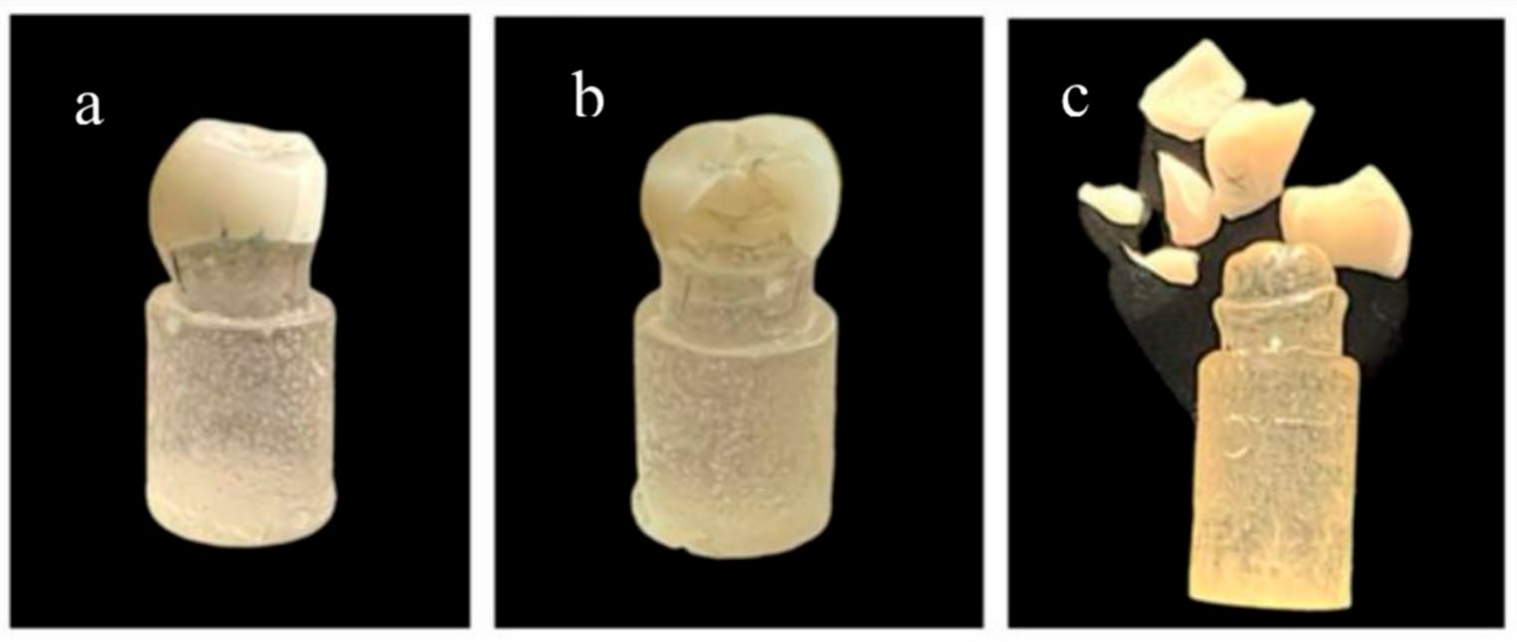
**a**, Cracking. **b**, Chipping and partial fracture. **c**, Catastrophic and fragments fracture

Failure modes were observed and assigned into 3 groups: Type I: Cracking (Fig. 2a), Type II: Chipping or partial fracture (Fig. 2b), and Type III: Catastrophic or fragmented fracture (Fig. 2c) as shown in Table 6.

**Fig. 3:**
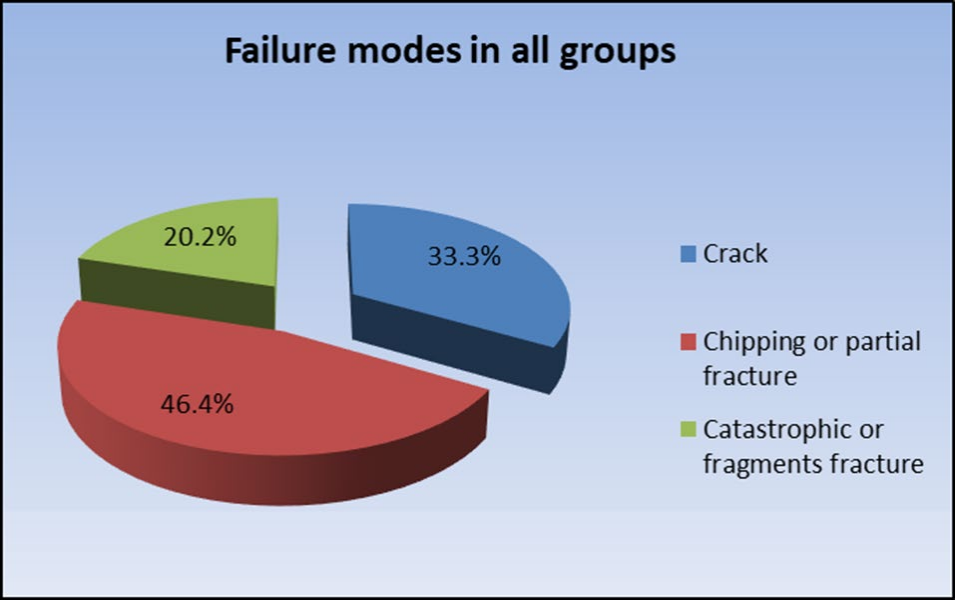
Pie chart representing percentage distribution of failure modes in all groups

The highest prevalence of cracks was found in groups; group CT0, group AT0 and group A T2. The highest prevalence of chipping or partial fracture was found in groups; group LT0, group ET0, group LT1, group LT1, group CT1 and group AT1. The highest prevalence of catastrophic or fragments fracture was found in groups: group LT2, group ET2 and group CT2 (Fig. 3).

**Table 6:** Frequencies (n), percentages (%) and results of Fisher’s exact test for comparison between failure modes in different groups

Group	Crack		Chipping or partial fracture		Catastrophic or fragments fracture		*P*-value	Effect size (v)
	*n*	%	*N*	%	*n*	%		
IPS e.max press Control	3	42.9	4	57.1	0	0	0.039*	0.466
LiSi press Control	3	42.9	4	57.1	0	0		
Celtra press Control	4	57.1	3	42.9	0	0		
VITA Ambria Control	5	71.4	2	28.6	0	0		
IPS e.max pressTempering − 5%	1	14.3	2	28.6	4	57.1		
LiSi press Tempering − 5%	0	0	3	42.9	4	57.1		
Celtra pressTempering − 5%	0	0	3	42.9	4	57.1		
VITA Ambria Tempering − 5%	4	57.1	2	28.6	1	14.3		
IPS e.max press Tempering − 9%	2	28.6	3	42.9	2	28.6		
LiSi press Tempering − 9%	2	28.6	4	57.1	1	14.3		
Celtra press Tempering − 9%	2	28.6	4	57.1	1	14.3		
VITA Ambria Tempering − 9%	2	28.6	5	71.4	0	0		
Total	28	33.3	39	46.4	17	20.2		

*: Significant at *P* ≤ 0.05

### Scanning electron microscope (SEM)

**Fig. 4:**
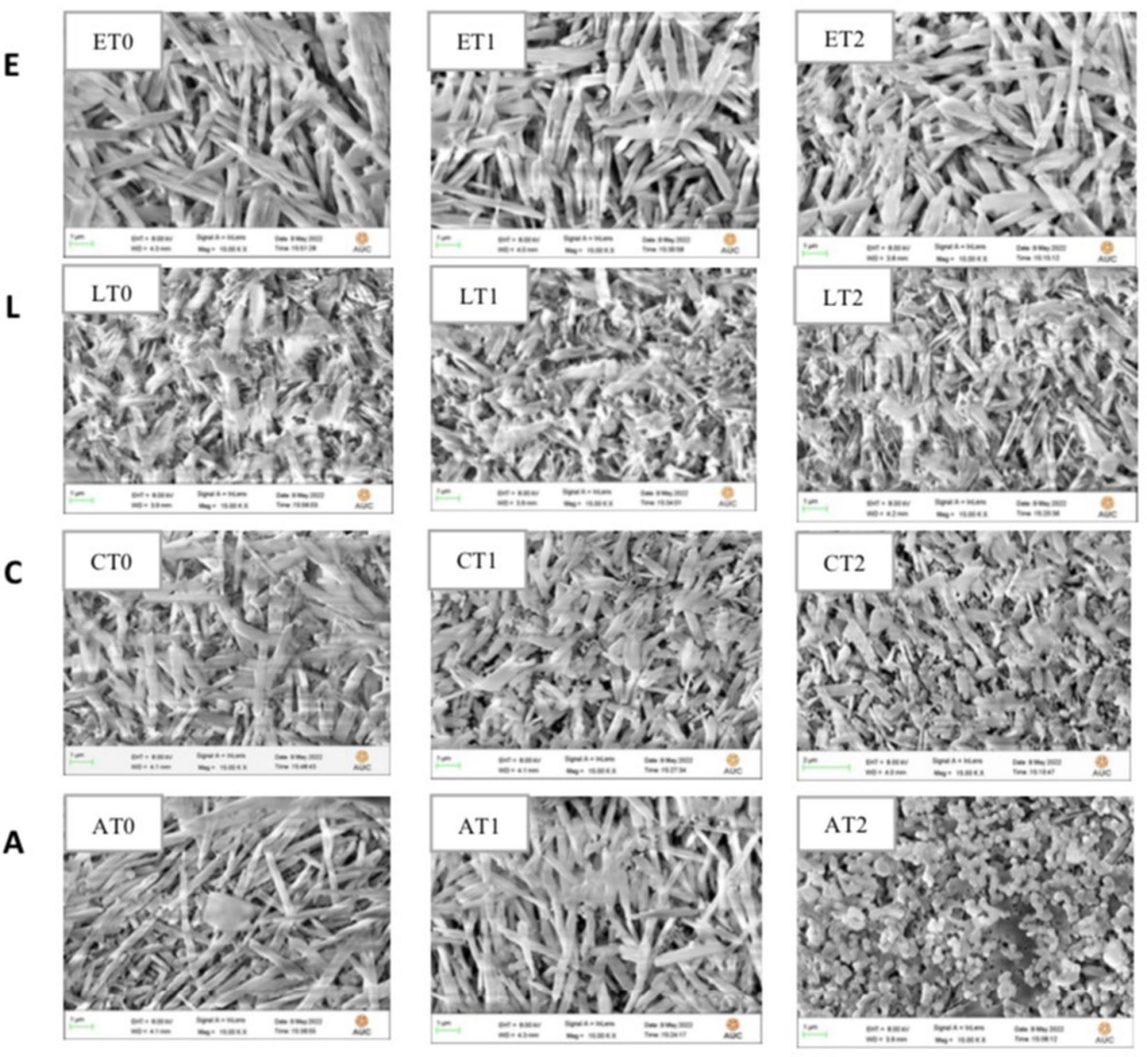
**E**, SEM image (15,000x) of IPS e.max press T0, T1 and T2. **L**, SEM images (15,000x) of LiSi press T0, T1 and T2. **C**, SEM images (15,000x) of Celtra press T0, T1 and T2. **A**, SEM images (15,000x) of Vita Ambria T0, T1 and T2

The SEM image observations of subgroup (ET2) showed broader, shorter and highly fused crystals than subgroups (ET0) and (ET1). The range of the crystal length was 1.305–1.758 μm, while the range of detected width was 695.0–871.2 nm (Fig. 4 ET0-ET2). The SEM image observations of group (LT2) showed elongated crystals and highly interlocking microstructure than subgroups (LT0) and (LT1). The crystals length was 2.299–2.403 μm while the width was 497.6–985.6 nm (Fig. 4 LT0-LT2). The SEM image observations of group (CT2) showed shorter crystals and highly fused together than group (CT0) and (CT1). The crystals length was 1.305–1.758 μm, while the width was 510.4–640.4 nm (Fig. 4 CT0-CT2). The SEM image observations of group (AT2) showed nano-shaped clusters, well-aggregated with each other and more fused together than than group (AT0) and (AT1). The width was 274.1–283.9 nm (Fig. 4 AT0-AT2).

### Xray diffraction test (XRD)

**Fig. 5:**
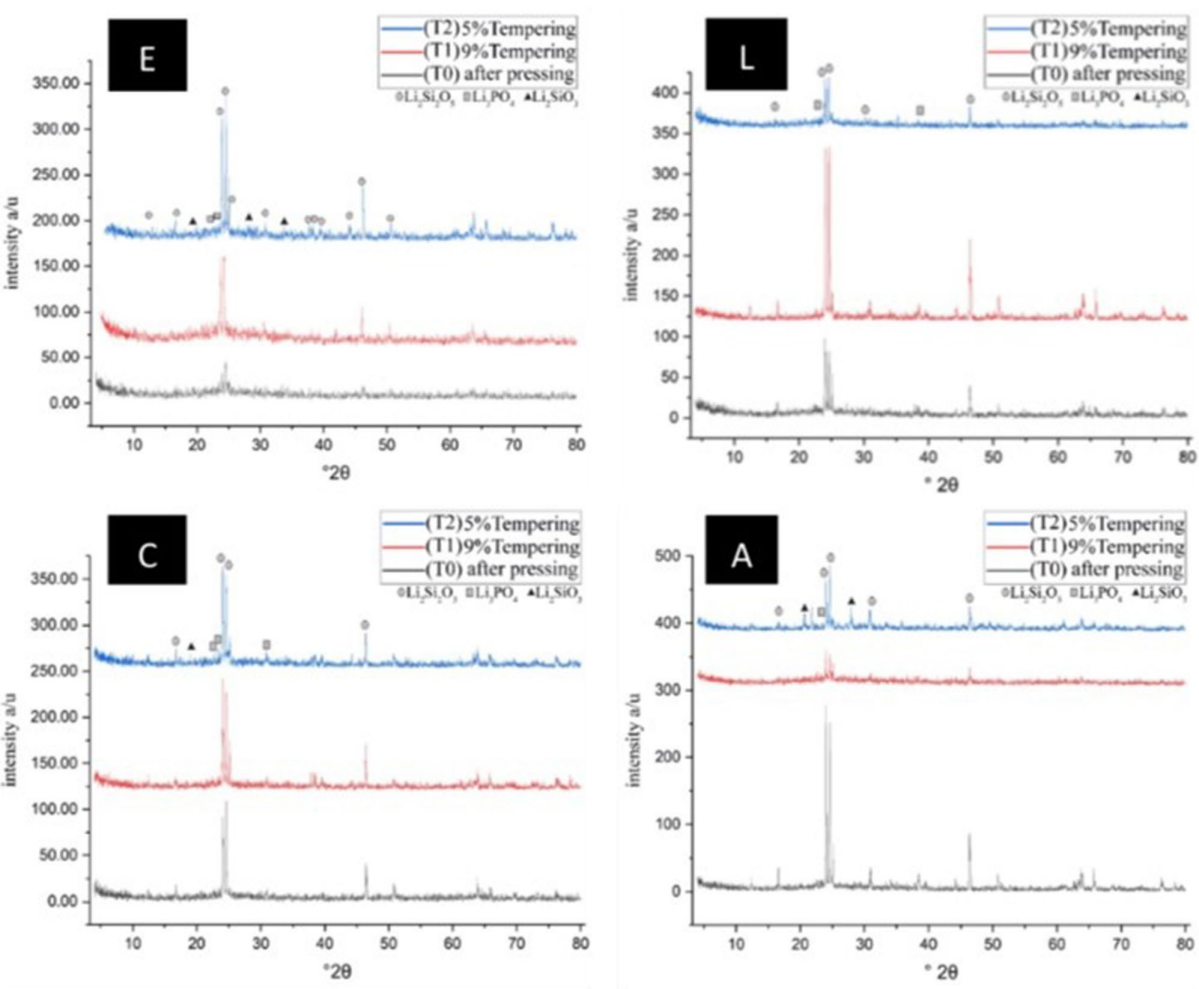
**E**, XRD analysis of of IPS e.max press T0, T1 and T2. **L**, XRD analysis of of LiSi press T0, T1 and T2. **C**, XRD analysis of of Celtra press T0, T1 and T2. **A**, XRD analysis of Vita Ambria T0, T1 and T2

The X-ray analysis (XRD) detected diffraction peaks that correspond to the present crystalline phases. Lithium disilicate was identified to be the main crystalline phase for IPS e.max press samples, Initial LiSi press samples and VITA Ambria samples. While lithium silicate was identified to be the main crystalline phase for Celtra press samples. Major peaks for Lithium disilicate (Li_2_ Si_2_ O_5_) were observed at 2θ values of 24.7 degrees, 24.2 degrees, and 40 degrees. Traces of lithium metasilicate (Li_2_ SiO_3_) and lithium phosphate (Li_3_ PO_4_) were also detected. The dominant peak (highest peak) was at 24.7 degrees The XRD data for Group (E) showed that the peaks of groups T1 and T2 were similar, the crystalline phase assemblage did not change; however, their radiation intensities had the highest peak for the tempered samples compared to the control samples. The unchanged crystalline phase was demonstrated for other ceramic groups, however the peak intensity for LiSi ceramics was the highest in T1 and the highest peak intensity was observed in the control samples for VITA Ambria. (Fig. 5).

### Energy dispersive x-ray analysis (EDAX)

The elemental surface composition of each subgroup is presented in (Table 5) for all groups.

It was revealed that the elemental surface composition was almost the same among subgroups as shown in Table 7.

**Table 7:** Microanalysis by EDAX of Four Heat Pressed Lithia-based glass ceramic

Elements	IPS e.max press (Mass %)			Initial LiSi press (Mass %)			Celtra press (Mass %)			VITA Ambria (Mass %)		
	T0	T1	T2	T0	T1	T2	T0	T1	T2	T0	T1	T2
O K	33.72	30.03	38.5	33.69	29.17	22.76	32.32	31.85	40.95	38.37	30.03	52.13
Na K	0.10	1.16	0.28	1.53	0.46	0.12	0.18	0.28	1.10	0.20	1.16	0.26
Al K	1.54	5.58	1.08	3.92	3.91	1.42	1.25	4.04	3.36	1.85	5.68	0.86
Si K	49.64	50.74	53.7	50.79	49.95	46.85	46.86	46.81	48.42	52.46	50.47	41.24
K K	1.46	2.26	2.36	2.60	6.66	0.92	0.91	1.70	1.61	2.54	2.26	1.20
Ca K	0.16	0.97	0.11	0.02	0.56	0.13	0.15	ND	0.10	0.21	0.97	0.06
Zn L	ND	ND	0.65	ND	1.79	ND	0.12	ND	0.28	0.40	ND	0.21
Zr L	7.03	2.97	ND	1.58	1.25	7.10	5.49	9.08	0.73	0.20	2.97	ND

**T0** Control group, **T1** Tempering 9% below pressing temperature and **T2** Tempering 5% below pressing temperature

## Discussion

This in-vitro study aimed to assess the fracture resistance of four heat pressed glass ceramics. As manufactures postulated that thermal tempering can increase the fracture resistance of lithia based glass ceramics, different thermal tempering protocol was selected in this study. One of the thermal tempering temperatures utilized in this research was calculated according to the manufacturer’s instructions of Vita Ambria which was 9% below pressing temperature and another intermediate thermal tempering temperature (5% below pressing temperature) was also selected.

The crowns in this study were cemented and tested over epoxy resin dies to obtain fracture resistance values near to those cemented on dentin. The modulus of elasticity of epoxy resin (12.9 GPa) is comparable to the reported modulus of elasticity of human dentin (14.7 GPa) [50–52].

According to the comparison between different ceramic types within each tempering temperature, the results of this study showed that IPS e.max press had the highest statistically significant mean fracture resistance value. This finding could be attributed to the microstructural features (SEM) of IPS e.max press specimens that displayed elongated spindle shaped crystals, sharp and pointed edges forming highly interlocking microstructure that led to increasing the fracture resistance.

Also, the higher pressing temperature of IPS e.max press (917 °C) might allow more crystal growth and interlocking. These results were in agreement with a study by Wang F et al. [53] who discussed the influence of various heat-pressing temperatures on the microstructure and flexural strength of lithium disilicate glass ceramic. They stated that heat-pressing temperature at 950 °C with a holding time of 15 min achieved almost pore-free microstructure and the highest flexural strength.

The results are in agreement with a study by Sieper K [31] who concluded that the mean fracture strength of lithium disilicate crowns showed a significantly higher fracture strength compared to zirconia reinforced lithium silicate crowns.

On the other hand, the results are inconsistent with a study by Donmez et al. [54] and Hamza et al. [55], who concluded that zirconia-reinforced lithium silicate (ZLS) had higher fracture resistance values than lithium disilicate after subjected to different aging processes.

Celtra press and VITA Ambria showed the lowest mean fracture resistance. This might be explained by the addition of ZrO_2_ as a nucleating agent that hindered crystal growth. Therefore, smaller lithium silicate crystalline phases were present in the pressed samples compared to ZrO_2_-free glass ceramics. This finding was supported by SEM images of this study where Celtra press showed shorter and flatter crystals with less interlocking and more inter crystal spaces. These smaller crystals adversely affected the mechanical properties of the glass-ceramic. These results were also in agreement with Apel E et al. [56] who stated that the incorporation of ZrO_2_ in the glass matrix did not increase the flexural strength. This was explained by the increase in viscosity due to the high ZrO_2_ content in the glass- ceramic and the associated reduction in the crystal growth of lithium silicate and lithium disilicate.

The null hypothesis in the present study was rejected as both ceramic type and thermal tempering had significant effect on the crowns’ fracture resistance. This could be attributed to the influence of the thermal tempering protocol on the crystal size and its morphology. This behavior is called “Ostwald ripening” according to Apel et al. [56], it takes place when the microstructure coarsens and liberates surface energy excess due to the solubility of small particles. As a consequence, larger crystals grow at the expense of smaller ones. Larger-sized crystals increase the interlocking effect which leads to increasing the fracture resistance. These results are in agreement with EL-Etreby et al. [22], Albakry et al. [2] and Gorman et al. [57].

This finding was supported by SEM images of this study where highly interlocking and broader crystals were presented in tempered samples resulting in the highest fracture resistance mean value. These results were in agreement with a study by Li D et al. [58], who evaluated the effect of crystal size on the mechanical properties of a lithium disilicate glass-ceramic. They stated that crystals with greater aspect ratios have been proposed to enhance the “interlocking effect” of the crystalline phase, benefiting the strengthening of the glass-ceramics.

For IPS e.max crowns: thermal tempering 5% below pressing temperature showed higher statistically significantly higher mean fracture resistance than 9% below pressing temperature and control group. This can be explained by the broader, shorter and highly fused crystals demonstrated in their SEM photos. The results are inconsistent with a study conducted by Tang et al. who concluded that flexural strength of IPS e.max press decreased when subjected to heat tempering and explained that by the increase in porosity after heat tempering.

In the present study the elemental surface compositions and phase analysis of both heat pressed and thermal tempering samples were the same. These results coincide with EL-Etreby et al. [2], Chung et al. [59] and Albakry et al. [2] Lithia based glass ceramic crowns used in this study can be thermally tempered to improve the fracture resistance without appreciably changing the crystalline composition.

On examination of fractured samples, it was clearly observed that the most common failure mode (cracks) was found in VITA Ambria thermally tempering at 5% below pressing temperature (T2). This finding could be attributed to the microstructural analysis (SEM) of Ambria control showed nano-clusters, well aggregated with each other and more fused together forming larger clusters. The fracture resistance results of crowns in the current study are clinically accepted within the mean reported biting force of molars 600–900 N [25]. Therefore, the tested crowns are able to withstand the maximum posterior masticatory forces and presented favorable modes of failure which were mostly cracks and chipping fractures.

### Limitations of the study

1. The current study is an in vitro assessment which need further research, particularly in clinical settings, to confirm the findings and understand the long-term effects of thermal tempering on the performance and aesthetic properties of lithia-based glass ceramic crowns in the human mouth.
2. The study did not consider long-term aging effects such as thermo-mechanical aging which could influence the fracture resistance and microstructure of the crowns.

## Conclusion

### Within the limitation of this in vitro study, the following could be concluded

1. Incorporation of zirconia oxide into the lithium disilicate glass matrix did not show improvement in the fracture resistance.
2. Thermal tempering procedure had a significant impact on fracture resistance.
3. Increasing the tempering temperature near the pressing temperature had a positive impact on the fracture resistance.
4. Thermal tempering technique had no influence the elemental surface composition and phase analysis. Thermal tempering 5% below pressing temperature showed changes in crystal size and orientation.

## Data Availability

All relevant datasets and their supporting information files generated and analyzed during this study are available from the corresponding author upon reasonable request.

## Footnotes

1 Core Team (2023). R: A language and environment for statistical computing. R Foundation for Statistical Computing, Vienna, Austria. URL https://www.R-project.org/.

## Abbreviations

C.N.C: Computerized Numerical Control
CAD/CAM: Computer Aided Design/Computer Aided Manufacture
EDAX: Energy Dispersive X-Ray Analysis
SEM: Scanning Electron Microscopy
XRD: Xray Diffraction
Li_2_Si_2_O_5_: Lithium Disilicate Crystals
ZLS: Zirconia Reinforced Lithium Silicate Ceramic
Gpa: Gigapascal
